# Atypical *Streptococcus suis* in Man, Argentina, 2013

**DOI:** 10.3201/eid2003.131148

**Published:** 2014-03

**Authors:** Raquel Callejo, Monica Prieto, Francisco Salamone, Jean-Philippe Auger, Guillaume Goyette-Desjardins, Marcelo Gottschalk

**Affiliations:** Instituto Nacional de Enfermedades Infecciosas, Buenos Aires, Argentina (R. Callejo, M. Prieto);; Hospital San Martín, Entre Ríos, Argentina (F. Salamone);; University of Montreal, St-Hyacinthe, Québec, Canada (J.-P. Auger, G. Goyette-Desjardins, M. Gottschalk)

**Keywords:** Streptococcus suis, bacteria, atypical strain, peritonitis, Argentina, Latin America

**To the Editor:** *Streptococcus suis* is a major swine pathogen and an emerging zoonotic agent that causes mainly meningitis and septic shock (*1*,*2*). Among the 35 described serotypes classified by differences in capsular antigens, serotype 2 is the most frequently isolated from humans worldwide, and serotype 14 cases are also increasing in some countries (*1*). In Southeast Asia, this pathogen affects not only workers in close contact with pig/pork by-products but also the general population, probably because of the widespread presence of backyard types of pig production, open meat markets, and some special dishes prepared with raw meat or blood (*3*). We report a case of peritonitis caused by an atypical *S. suis* serotype 21 strain in a patient in Argentina.

A 62-year-old man from Santa Fe Province in Argentina, who had a history of tobacco and alcohol abuse, was hospitalized in 2013 as an emergency patient with symptoms of acute abdominal distress. Ten days before admission, abdominal distention, accompanied by intense upper abdominal pain, developed in the patient. The patient’s family reported that he had been having gastrointestinal bleeding 4 days before admission, and he was suspected of having diabetes.

At admission, a physical examination indicated jaundice, hepatosplenomegaly, and ascites. A neurologic examination indicated that the patient was conscious, but disoriented, and that his vital signs were stable. The patient had a temperature of 38.9°C, a pulse rate of 130 beats/min, and blood pressure of 110/70 mm Hg. Other laboratory results were a leukocyte count of 2,900 cells/μL (70% neutrophils), a platelet count of 94,000/μL, a serum hemoglobin concentration of 13.20 g/dL, a glucose concentration of 195 mg/dL, a blood urea nitrogen level of 42 mg/dL, a creatinine level of 0.96 mg/dL; a serum bilirubin level of 3.01 mg/dL, an alanine aminotransferase level of 35 U/L, an aspartate aminotransferase level of 70 U/, a serum albumin level of 2.66 g/dL, and an increase in prothrombin time to 22 s.

Spontaneous bacterial peritonitis was suspected. Abdominal paracentesis was performed and produced a turbid milky fluid, with a protein level of 1600 mg/dL; 1,340 cells/µL (90% neutrophils), a lactate dehydrogenase level of 221 U/L, and an amylase level of 34 U/L. Samples of blood and ascitic fluid were inoculated into aerobic and anaerobic blood culture bottles. Gram staining was performed and no organisms were observed.

Treatment with intravenous ceftriaxone (2g/day) was started after a diagnosis of spontaneous bacterial peritonitis associated with liver cirrhosis was made. After 48 h of incubation, cultures of blood and ascetic fluid were plated onto sheep blood agar and chocolate agar and incubated at 35°C in an atmosphere of 5% CO_2_. After 24 h of incubation, cultures showed growth of α-hemolytic streptococci.

An API Strep Test (bioMérieux, Marcy l’Etoile, France) identified the isolate as *S. pneumoniae* (probability 58.7%) or *S. suis* (probability 20.7%). However, these 2 probability values are unacceptable identification confidence levels. Therefore, the species and serotype were identified by sequence analysis of a 16S rRNA gene and a coagglutination test as described (*4*,*5*). The isolate was identified as *S. suis* serotype 21.

The infection was considered resolved when all signs and symptoms of infection disappeared, a polymorphonuclear cell count in ascitic fluid decreased to <250 cells/μL, and ascitic fluid cultures were negative for bacteria. Antimicrobial drug therapy was given for 48 h after resolution of the infection. The patient denied any recent occupational or occasional contact with swine or other animals, and he had no history of eating raw or undercooked pork.

A biochemically and antigenically atypical strain was isolated from the patient with peritonitis. A reference strain of serotype 21 and most other strains of this serotype had been isolated from tonsils of healthy pigs (*6*). However, 16 strains had also been isolated from sick pigs during 2008–2011 in Canada (*7*). These findings indicate that this serotype is potentially virulent. Most strains, including the strain from the patient reported, are usually not identified as *S. suis* by rapid multitest identification systems (*6*).

There are only 2 reports of *S. suis* being isolated from humans in Latin America; these reports were also from Argentina (*8*,*9*). Because swine production in Argentina is a smaller industry than in other Latin American countries, the higher rate of *S. suis* isolation rate is probably the consequence of good surveillance systems and awareness of the pathogen by local diagnostic laboratories.

The patient did not have any contact with swine, pork-derived products, or raw/undercooked beef. A patient infected with *S. suis* might be unaware or have no recollection of exposure to animals. Latent infection, with reactivation many years later, has been reported (*10*). *S. suis* might become an opportunistic pathogen in persons who are stressed or immunodeficient. This pathogen has also been increasingly isolated from mammals other than pigs and from the environment. The patient in this study had a history of alcohol consumption, which is a reported risk factor for this infection (*3*).
